# WHOLE-BODY MRI TO ASSESS SUBCLINICAL CARDIOVASCULAR DISEASE AND FRAILTY DEVELOPMENT

**DOI:** 10.1093/geroni/igz038.336

**Published:** 2019-11-08

**Authors:** Bharath Ambale-Venkatesh, Jaclyn Sesso, Jeremy Walston, Karen Bandeen-Roche, Joao Lima

**Affiliations:** 1 Johns Hopkins University, Baltimore, Maryland, United States; 2 Johns Hopkins Bloomberg School of Public Health, Baltimore, Maryland, United States

## Abstract

Cardiovascular disease (CVD) and frailty have been linked at the mechanistic and epidemiological levels. Our goal is to identify if subclinical markers such as atherosclerosis, body composition, and fibrofatty infiltration measured from non-contrast whole-body magnetic resonance imaging (MRI) are markers of physical frailty. Community dwelling older adults with frailty status ascertained by Fried measurement are being recruited from an aging studies registry. MRI is performed using a Canon Galan3T with dedicated coils. Preliminary analysis from 4 frail individuals (86±15 years, 3 female, BMI=22±3kg/m2) and 2 age-matched robust controls (86±1 years, 1 female, BMI=28±0.2kg/m2) is presented. Of 4 frail one had a prior heart attack; one was previously diagnosed with heart failure. Mean atheroma score from 28 vessel segments (0.42±0.26 vs 0.18±0.10) and aortic tortuosity (2.3±0.4 vs 2.1±0.1) were higher in frail compared to robust indicative of higher atherosclerotic burden and vascular stiffness. Mean subcutaneous and visceral adipose tissue volumes were lower in frail compared to robust. However, mean myocardial (1113±27 vs 1089±2), liver (729±92 vs 683±104) and skeletal muscle (1106±25 vs 1072±64) T1 times (milliseconds) were each higher indicative of greater diffuse interstitial fibrosis. Averaged intramuscular fat percent measured across the pelvis, forearm, pectus, thigh, and calf was higher in frail compared to robust (14.8±4.1% vs 8.5±2.3%) indicative of higher fatty infiltration. Although these early results do not reach statistical significance, they support further study to determine cardiovascular and tissue related differences between physically frail and robust older adults, which in turn may inform intervention developments for frailty and CVD.

